# Impact of COVID-19 on women living with HIV who are survivors of intimate partner violence

**DOI:** 10.1186/s12889-024-18862-7

**Published:** 2024-05-20

**Authors:** Xinyi Zhang, Carolina R. Price, Alexandrya S. Pope, Tami P. Sullivan, Jaimie P. Meyer

**Affiliations:** 1 Chronic Disease Epidemiology, Yale School of Public Health, New Haven, CT USA; 2 Section of Infectious Diseases, Yale School of Medicine, 135 College Street, Suite 323, New Haven, CT 06510 USA; 3 Department of Psychiatry, Yale School of Medicine, New Haven, CT USA

**Keywords:** COVID-19, HIV, Women, Intimate partner violence

## Abstract

**Background:**

Women living with HIV (WLWH) experience higher rates of intimate partner violence (IPV) compared to women without HIV, but there has been minimal research to date on the impact of the COVID-19 pandemic on the lived experiences of WLWH who are IPV survivors.

**Methods:**

This is a secondary analysis of COVID-19 impact using baseline data from an ongoing, prospective, micro-longitudinal cohort study of HIV care engagement among WLWH who have experienced lifetime IPV. We measured the impact of COVID-19 along key domains (i.e., physical health, day-to-day life, sexual/relationship behavior, substance use, HIV care, mental health, financial status, and having conflict with partners). Using independent t-tests or Fisher’s exact tests, and Pearson’s chi-squared tests, we compared women with and without ongoing IPV across sociodemographic characteristics, psychiatric disorders, substance use, and COVID-19 impact domains. We then built separate multivariate linear regression models for each of the different COVID-19 impact domains; ongoing IPV exposure was the primary explanatory variable of interest.

**Results:**

Enrolled participants (*n* = 84) comprised a group of women (mean age 53.6y; SD = 9.9) who were living with HIV for a mean 23.3 years (SD = 10), all of whom had experienced lifetime IPV. Among 49 women who were currently partnered, 79.6% (*n* = 39) reported ongoing IPV. There were no statistically significant differences between those experiencing ongoing IPV and those who were not (or not partnered) in terms of demographic characteristics, substance use, or mental health. In multivariate models, ongoing IPV exposure was not associated with any COVID-19 impact domain. Anxiety and depression, however, were associated with COVID-19-related physical health, HIV care, and relationship conflict. Hispanic ethnicity was significantly associated with COVID-19-related physical health. More severe cocaine and opioid use were also significantly associated with COVID-19-related impact on day-to-day life.

**Conclusions:**

Among this sample of WLWH who are all lifetime IPV-survivors, nearly half had ongoing IPV exposure. The COVID-19 public health emergency period affected WLWH in varied ways, but impacts were most profound for women experiencing concurrent mental health and substance use problems. Findings have important implications for future interventions to improve women’s health and social outcomes.

**Supplementary Information:**

The online version contains supplementary material available at 10.1186/s12889-024-18862-7.

## Background

Nearly 1 in 2 women in the United States report experiencing intimate partner violence (IPV) in their lifetimes, including contact sexual violence, physical violence, and/or unwanted pursuit victimization by an intimate partner [1]. Emerging global data suggests that there was an increase in IPV during the height of the COVID-19 pandemic beginning in March 2020 [2], including in China [3], France, and Argentina [4]. In the U.S., ‘stay home’ regulations during 2020 were associated with an 8% increase in reported domestic violence incidents [5]. These data were mainly based on crime or hotline data, which likely underestimated IPV victimization because many women who experience IPV do not report it [6]. To date, few studies have examined IPV experiences during the COVID-19 public health emergency period using self-reported data or validated behavioral measures [7–11]. 

Women living with HIV (WLWH) experience IPV at a rate 12–32 times higher than women nationally [12, 13]. IPV can directly and indirectly affect women’s physical, sexual, psychological, and reproductive health [14] and, for WLWH, IPV also can be associated with reduced engagement in HIV care and antiretroviral adherence, leading to lower likelihood of HIV viral suppression [13, 15, 16]. WLWH likely faced additional health- and IPV-related stressors during COVID-19. In the height of pandemic-related restrictions in many U.S. settings, HIV healthcare transitioned to virtual or telephone-based visits [17]. Lockdowns were also isolating for some women, such that WLWH experiencing IPV may have been less able to connect to their community’s critical social and protective networks [17, 18]. 

The purpose of this study is to fill an important gap in our understanding about IPV exposure among WLWH during the COVID-19 pandemic, using self-reported data with validated IPV measures. We broadly evaluated COVID-19 impact on physical health, day-to-day life, sexual/relationship behavior, substance use, HIV care engagement, mental health, financial/economic status, and partner conflict. Beyond describing IPV exposure and COVID-19 pandemic impact among WLWH, our hypothesis was that COVID-19 affected WLWH differently based on whether their IPV exposure was ongoing or in the past- that women experiencing ongoing IPV would be more affected by the COVID-19 pandemic across various domains compared to women not experiencing ongoing IPV. This analysis is needed to disentangle how the context of the COVID-19 public health emergency influenced the health and social outcomes of WLWH.

## Methods

This is a cross-sectional secondary data analysis of COVID-19 impact and experiences of IPV among WLWH using baseline data from an ongoing, prospective, micro-longitudinal study. Micro-longitudinal designs (that include ecological momentary assessments) involve near-real time data collection, for example using daily surveys or diaries [19]. The purpose of the parent study is to understand how exposure to IPV affects women’s day-to-day experiences of living with HIV, including adherence to antiretroviral therapy (ART), engagement in HIV care, and HIV viral suppression. Participants engage in a baseline study interview, 32 days of twice-daily data reporting, and subsequent interviews at 6 months following baseline. The study is observational and there is no intervention component, so it does not meet criteria for a clinical trial and is not registered as such. Further details on the parent study may be found on NIH RePORTER (https://reporter.nih.gov) for R01MH121991. Study enrollment began in April 2021 and is ongoing. Reporting on primary outcomes is forthcoming once data collection is complete.

### Study sample

Participants are being recruited from local HIV care clinics and other community-based organizations (CBOs) that serve WLWH (e.g., AIDS service organizations, federally qualified health centers, Ryan White-funded HIV clinics, peer support services, and case management agencies). Recruitment materials are posted in CBO lobbies and clinic rooms, and on social media through Facebook (Meta) ads that are restricted to adult women in the Greater New Haven area. Multiple outreach methods are utilized: (1) research assistants are onsite at HIV clinics weekly to meet with potentially interested patients and screen for eligibility; (2) WLWH can self-refer using a QR code to a secure Qualtrics link that is printed on posted promotional material, or contact the study team directly through a dedicated study phoneline or email; (3) healthcare providers can directly refer WLWH who express interest in learning more about the study through a Best Practices Advisory in the electronic health record; or (4) enrolled participants can refer their peers using an incentivized modified respondent driven sampling strategy that we have previously used [20]. All referral strategies collect only basic contact information and preferred method of contact, with the priority of protecting participant safety and privacy. Trained research assistants screen referred individuals for the following criteria: adults (18 years and older) who identify as women (i.e., cis- or trans-), are living with diagnosed HIV, and report any lifetime exposure to physical, sexual, and/or psychological violence in an intimate relationship. Women are ineligible to participate if they have experienced significant psychiatric instability based on self-reported inpatient psychiatric hospitalization in the past 6 months, are not comfortable conversing in English or Spanish, or have a legal conservator of person.

### Study procedures

Individuals who meet all eligibility criteria are offered enrollment and undergo written informed consent procedures. All procedures are approved by Yale University Human Investigations Committee (IRB). Following enrollment, all participants complete an in-person baseline study interview with a bachelor’s or master’s degree-level trained research assistant in a private research office. The interview takes approximately 3 h and participants are compensated $50 for their time. All baseline interview data are entered by the research assistant into REDCap electronic data collection software as the interview is being conducted [21]. The current analysis used only baseline data from the first 84 participants who were enrolled between April 2021 and June 2022. All baseline data were extracted from REDCap, deidentified, and exported into csv files for data cleaning and analysis.

### Measures

#### COVID-19 impact

The primary outcome for this analysis is COVID-19 impact. COVID-19 impact is assessed across 8 different domains: physical health, day-to-day life, sexual behaviors, substance use, HIV care, mental health, financial status, and having conflict with a partner, using a brief survey that we developed and have used to describe COVID-19 impact in other populations (Appendix 1) [22–24]. Participants are asked, “How much has COVID-19 directly affected…” for each domain, responding on a Likert scale of 1 (not at all) to 5 (extremely). We also ask, “How has COVID-19 affected you in terms of…” for each domain where participants can select all that apply from a list of options, including “other” with the option of a free-text response.

#### IPV exposure

All enrolled participants have experienced IPV in their lifetimes. The primary explanatory variable of interest is ongoing IPV exposure. Type of current IPV exposure is only assessed for women who were partnered in a relationship in the 30 days prior to study enrollment or the most recent HIV clinic visit.

*Physical IPV* is measured with the Revised Conflict Tactics Scale-2 (CTS-2) across 12 items in subscales for physical assault (α = 0.86), injury (α = 0.96), and negotiation (α = 0.86) in an intimate relationship [25]. Injury is defined as “partner-inflicted physical injury” causing bone or tissue damage, warranting medical attention, or causing pain for a day or more; and negotiation is the “action to settle a disagreement through discussion.” The cognitive subscales of negotiation are “examples of such discussions” whereas the emotion subscale measures “the extent to which positive affect is communicated.” [25] Response options referring to the 30 day period are: *0 = never; 1 = once; 2 = twice; 3 = 3–5 times; 4 = 6–10 times; 5 = 11–20 times; 6 = more than 20 times; 7 = not in those 30 days, but it happened before in our relationship.* Responses are recoded to the midpoint of the range of scores and are transformed using standardized syntax and categorized into type of physical IPV victimization, frequency and severity [26, 27]. *Sexual IPV* is measured by the Sexual Experiences Survey (SES), using the 10 items that classify and measure degrees of sexual victimization (α = .74 for women), using the same response options as for the CTS-2 as above [28, 29]. Variables are transformed using standardized syntax to calculate any sexual violence exposure in one’s lifetime [29]. *Psychological IPV* is measured using the Psychological Maltreatment of Women Inventory Short Version (PMWI-S), a 14-item instrument designed to assess the level of psychological abuse of women by their intimate male partners including subscales for dominance/isolation (α = .88) and emotional/verbal (α = .92) abuse [30]. Participants are asked how frequently they have experienced these things in the past 30 days; response options are: *1 = never; 2 = rarely; 3 = occasionally; 4 = frequently; 5 = very frequently.* Total PMWI score ranges 14–70 and each of the type sub-scores range 7–35, with higher scores indicating higher severity of psychological abuse.

We use the Past Abusive Behavior Inventory (also known as Past Abusive Relationships; PAR) to measure the number of past adult relationships in which women experienced psychological, physical, or sexual IPV [31, 32]. In addition, participants who are currently in a relationship or were in a relationship in the 30 days prior to the baseline interview are asked if they experienced minor physical, severe physical, sexual, psychological, or monitoring violence with that partner, using the same response options as for the PMWI-S as above. Current IPV exposure is defined as > 1 on any of these 5 items; no current IPV exposure (i.e., past lifetime IPV exposure only) is defined as 1 on all 5 items or not currently partnered.

#### Sociodemographic and health characteristics

We assess participant age, gender identity, ethnicity/race, sexual orientation, education level, housing, employment, income level, relationship status, basic sociodemographic characteristics of their current partners, health insurance status, years since HIV diagnosis, and usual frequency of visiting an HIV care provider.

#### Mental health

We assess for depression symptoms using the Center for Epidemiological Studies-Depression Scale (CES-D; α = 0.85) [33]. Scores range 0–60, with higher scores indicating greater severity of depressive symptoms, and are dichotomized at < 16 vs. ≥16, with the latter indicating clinically significant depressive symptoms or probable depression. We assess for anxiety using the Generalized Anxiety Disorder instrument (GAD-7; α = 0.85), which consists of 7 items [34]. Each of the 7 items is scored 0–3, and the total score ranges 0–21; scores are dichotomized at < 10 vs. ≥10, with the latter indicating probable generalized anxiety disorder. We assess PTSD symptom severity using the Posttraumatic Diagnostic Scale for DSM-5 (PDS-5; α = 0.95) [35], across 4 domains of PTSD: (1) re-experiencing; (2) avoidance; (3) negative alterations in cognition and mood; and (4) hyper-arousal. Each of the 20 items is scored 0–4, where *0 = not at all; 1 = once a week or less/a little; 2 = 2 to 3 times a week/somewhat; 3 = 4 to 5 times a week/very much; 4 = 6 or more times a week/severe*, and the PDS-5 total score ranges 0–80. Scores of 28–80 reflect probable diagnosis of PTSD.

#### Substance use

We assess alcohol use with the Alcohol Use Disorders Identification Test (AUDIT; α = 0.93) [36]. Total scores are dichotomized at < 8 vs. ≥8, with the latter indicating hazardous and harmful alcohol use [37]. We use the NIDA-Modified Alcohol, Smoking, and Substance Involvement Screening Test (NM-ASSIST) to assess use of criminalized drugs or prescription drugs for “non-medical reasons”, including cannabis (α = 0.85), cocaine (α = 0.91), prescription stimulants (k = 0.74), methamphetamine, inhalants, sedatives (α = 0.87), hallucinogens, “street opioids”, and prescription opioids (α = 0.85) [38, 39]. “Non-medical reasons” for substance use are defined as *taking medications for reasons or in doses other than prescribed to you*. For each substance, participants are asked about past 3-month use frequency and substance use disorder criteria; substance involvement scores are summed to reflect current substance-specific severity, or an estimate of an individual’s risk for problems associated with that substance [38]. Scores 0–3 are categorized as lower severity; 4–26 as moderate severity, and scores ≥ 27 suggest high severity drug use; levels are used to identify appropriate interventions. Participants are also asked about the use of medications for the treatment of opioid use disorder.

### Statistical analysis

We used descriptive statistics to characterize the study sample. Continuous measures are presented as means with standard deviations or medians with interquartile ranges (IQR) if not normally distributed, and categorical measures as frequencies with proportions. To evaluate how the COVID-19 pandemic impacted women differently based on whether they experienced ongoing IPV, we compared participants experiencing current/ongoing IPV to participants not experiencing current/ongoing IPV (past IPV only) in terms of sociodemographic, mental health, and substance use characteristics, using independent t-test or Fisher’s exact test for continuous variables, and Pearson’s chi-squared test for categorical variables. Next, we explored associations between ongoing IPV exposure and different types of COVID-19 impact (physical health, day-to-day life, sexual/relationship behaviors, HIV care engagement, mental health, and having conflict with a partner). We did not build separate models for substance use or financial/economic COVID-19 impact domains because most women reported no impact of COVID-19 in terms of these factors, so there was insufficient variability to allow for generation of meaningful models. Otherwise, we conducted multiple linear regression analyses for each COVID-19 impact domain. The primary explanatory variable of interest was ongoing IPV; other included explanatory variables were presence of mental health problems and substance use. We also included sociodemographic variables (age, race, ethnicity, years of education, and employment status) as potential covariates. We developed full models of COVID-19 impact that included IPV exposure, age, race, ethnicity, years of education, employment status, PTSD, anxiety, depression, and substance use severity (for alcohol, cannabis, cocaine, opioids). Only variables with *p*-value < 0.2 in the full model are included in the reduced model. If co-linearity was plausible and supported by cross-tabulation of the data, only the variable that was more strongly associated with COVID-19 impact was retained in multivariable models. For the final, reduced model, statistical significance was defined as a *p*-value < 0.05. All analyses were performed using SAS (SAS 9.4, SAS Institute, Inc., Cary, NC).

## Results

### Baseline characteristics by IPV exposure recency

Eighty-four women (including 79 cis- and 5 trans-gender women) were enrolled and included in this analysis. As shown in Table 1, participants ranged from 23 to 75 years of age, with a mean age of 53.6 (SD = 9.9) years. The sample was racially/ethnically diverse with more than two-thirds (69.1%) identifying as Black/African American and 22.6% identifying as Hispanic/Latina. Most participants had a high school education (with 11.8 mean years of formal education; SD = 1.9) and experienced unemployment (84.5%). Participants had been living with diagnosed HIV for a mean of 23.3 years (SD = 10).

Mental health problems were highly prevalent: 31.0% (*n* = 26) met the threshold for probable PTSD diagnosis, 27.4% (*n* = 23) screened positive for generalized anxiety, and over half (52.4%; *n* = 44) had clinically significant depression symptoms. Substance use was common, including 14% (*n* = 12) meeting criteria for hazardous drinking. Most (73.8%) participants reported cannabis use and half (50%) had moderately severe cannabis use. Among the participants who used cocaine (*n* = 58), over half had moderate or high severity cocaine use. Of the participants who used street opioids (*n* = 24), nearly 80% had moderately severe opioid use. Additionally, 16 participants reported the use of prescription opioids and over half of them (56.3%) had moderately severe use of prescription opioids.

Given study inclusion criteria, all WLWH in the sample experienced some form of lifetime IPV, and of the 49 (58.3%) women currently in a relationship, 39 (79.5%) reported ongoing IPV exposure. As shown in Table 1, there were no significant differences between those experiencing ongoing IPV (*n* = 39) and those not experiencing ongoing IPV or not partnered (*n* = 10 and *n* = 35, respectively) in terms of any sociodemographic characteristics, mental health problems, or substance use.

### Current IPV exposure type and severity

Among the 49 currently partnered participants, almost half (44.9%) experienced physical assault, including minor physical assault (42.9%) and severe physical assault (28.6%). Fourteen (28.6%) were injured during conflicts with partners, 26.5% (*n* = 13) of whom experienced a minor injury, and 14.3% (*n* = 7) experienced a severe injury. Most currently partnered participants (95.9%) used negotiation strategies at some point in the last 30 days to deal with conflicts, including emotional negotiation (95.9%) and cognitive negotiation (93.9%).

### Correlates of COVID-19 impact

**Fig. 1:**
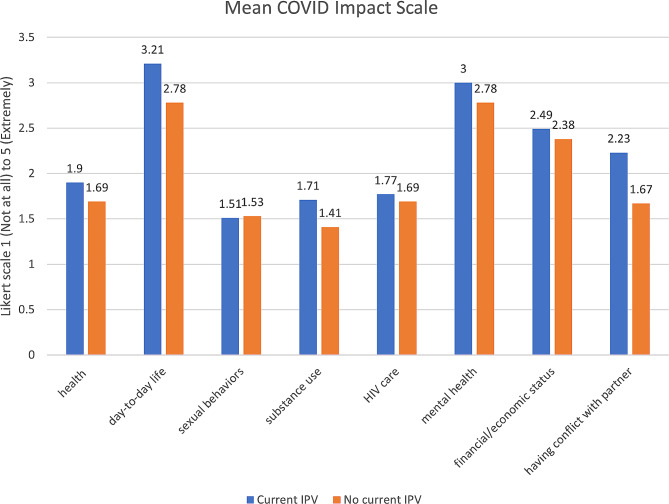
Mean COVID-19 impact scales (ongoing IPV vs. no ongoing IPV)

As shown in Fig. 1, COVID-19 had the greatest impact on women’s mental health and the least impact on sexual behaviors. Across all domains, mean COVID-19 impact scales (that ranged from 1 to 5) were higher in those experiencing ongoing IPV than among those who were not experiencing ongoing IPV, though the differences were not statistically significant.

Next, we turned to developing separate multivariate linear regression models for each of six COVID-19 impact domains. Contrary to our original hypothesis, after controlling for other key demographic, mental health problems and substance use in multivariable models, we found that ongoing IPV exposure was not significantly associated with any of the COVID-19 impact domains.

Participants reported a range of COVID-19-related physical health impacts, including experiencing symptoms of COVID-19 but not testing (*n* = 4); testing negative (*n* = 63); testing positive (*n* = 11); being exposed (*n* = 21); and being hospitalized for COVID-19 (*n* = 3). As shown in Appendix Table 1, the mean physical health impact score for women of Hispanic ethnicity was 0.927 points higher than for women who were not Hispanic (*p* = 0.007). WLWH who met the threshold for generalized anxiety were 0.537 points lower on the health impact scale than those who did not meet criteria for generalized anxiety, though this difference was not statistically significant (*p* = 0.144). WLWH with clinically significant depression symptoms scored 1.14 points higher on COVID-19 physical health impact than those without clinically significant depression (*p* = 0.0006).

**Table 1:** Baseline socio-demographic characteristics of women living with HIV (*N* = 84), by ongoing intimate partner violence experience

	*N* (%)	Ongoing IPV experience		*P*-value
		Yes (*N* = 39)	No (*N* = 45)	
Age (Mean ± SD)	53.58 ± 9.91	52.00 ± 9.54	54.95 ± 10.13	0.176
Gender identity				0.659
Cis-women	79 (94.05)	36 (92.31)	43 (95.56)	
Trans-women	5 (5.95)	3 (7.69)	2 (4.44)	
Ethnicity				0.097
Non-Hispanic or Latina	65 (77.38)	27 (69.23)	38 (84.44)	
Hispanic or Latina	19 (22.62)	12 (30.77)	7 (15.56)	
Racal background				0.847
Black/African American	56 (69.05)	28 (71.79)	30 (66.67)	
White/Caucasian	15 (17.86)	6 (15.38)	9 (20.00)	
Other	11 (13.10)	5 (12.82)	6 (13.33)	
Years of education (Mean ± SD)	11.79 ± 1.88	11.41 ± 2.34	12.11 ± 1.32	0.103
Employment status				0.218
Employed	13 (15.48)	4 (10.26)	9 (20.00)	
Unemployed	71 (84.52)	35 (89.74)	36 (80.00)	
In a relationship				< 0.001
Yes	49 (58.33)	39 (100)	10 (22.22)	
No	35 (41.67)	0	35 (77.78)	
Years of living with diagnosed HIV (Mean ± SD)	23.29 ± 10.00	22.82 ± 10.64	23.69 ± 9.52	0.694
PTSD				0.127
Probable PTSD diagnosis	26 (30.95)	16 (42.11)	10 (25.64)	
No probable PTSD diagnosis	51 (60.71)	22 (57.89)	29 (74.36)	
Missing	7 (8.33)			
Anxiety Screen				0.411
Generalized anxiety	23 (27.38)	13 (34.21)	10 (25.64)	
No generalized anxiety	54 (64.29)	25 (65.79)	29 (74.36)	
Missing	7 (8.33)			
Depression Screen				1
Clinically significant depression	44 (52.38)	22 (57.89)	22 (57.89)	
No clinically significant depression	32 (38.10)	16 (42.11)	16 (42.11)	
Missing				
	8 (9.52)			
Alcohol Use				0.133
Hazardous drinking	12 (14.29)	10 (47.62)	2 (16.67)	
Non-hazardous drinking	21 (25.00)	11 (52.38)	10 (83.33)	
Missing	51 (60.71)			
Cannabis use	N = 62	N = 32	N = 30	0.611
Lower severity	31 (50.0)	15 (46.88)	16 (53.33)	
Moderate severity	31 (50.0)	17 (53.13)	14 (56.67)	
Cocaine use	N = 58	N = 31	N = 27	0.709
Lower severity	21 (36.21)	10 (32.26)	11 (40.74)	
Moderate severity	33 (56.9)	18 (58.06)	15 (55.56)	
High severity	4 (6.90)	3 (9.68)	1 (3.7)	
Non-prescription opioids use	N = 24	N = 16	N = 8	0.726
Lower severity	4 (16.67)	2 (12.50)	2 (25.0)	
Moderate severity	19 (79.17)	13 (81.25)	6 (75.0)	
High severity	1 (4.17)	1 (6.25)	0	

Appendix Table 2 shows full and reduced models of COVID-19-related impact on day-to-day life. Women who identified as white reported lower COVID-impact on day-to-day-life compared to women who identified as Black or African American (*p* = 0.046). Compared to women with less severe cocaine use, WLWH with moderate/high severity cocaine use reported lower (though not statistically significant) COVID-19-related impact on day-to-day life (*p* = 0.076). Women with moderately/high severity use of street opioids and prescription opioids experienced a greater COVID-related impact on their day-to-day life than women with less severe opioid use.

Participants reported that COVID-19 impacted sexual/relationship behaviors in terms of reduced close contact (*n* = 13) or in-person dating (*n* = 5) and more frequently using barrier protection, like condoms or dental dams, (*n* = 4). Of the 7 participants who reported “other”, 2 specified: *partner hasn’t felt comfortable with having sex as often*; and *being more careful with other partners.* In the full model of COVID-impact on sexual behavior, only ethnicity was significant in that women who were Hispanic had a 0.734 (SE 0.487) point higher score compared to women who were not Hispanic (*p* = 0.137).

Women reported that COVID-19 affected how they engaged in HIV care, for example they reported: requesting a 90-day supply of medications (*n* = 8) or initiating home delivery of medications (*n* = 7), restarting HIV medications (*n* = 1), cancelling appointments (*n* = 12) and requesting telehealth visits (*n* = 14) during COVID-19. Additionally, some women reported difficulty accessing their pharmacy (*n* = 8), lab testing (*n* = 11) or other testing services (*n* = 14), and appointment scheduling (*n* = 22) during COVID-19. Two participants specified other impacts that included: *using drive thru, made sure to take HIV meds every day, doctor made telehealth appointments, contacted doctor about the COVID-19 vaccine.* As shown in Appendix Table 3, women who met the threshold for anxiety reported a greater impact of COVID-19 on their HIV care than women who did not (*p* = 0.030). Among WLWH using street opioids, those with moderate/high severity use reported greater impact of COVID-19 HIV care than those with lower severity opioid use (0 = 0.044). Associations between other mental health and substance use factors and COVID-19 impact on HIV care were not statistically significant in reduced models.

Participants reported that COVID-19 impacted their mental health in negative ways, including increased frustration or boredom (*n* = 41), greater anxiety (*n* = 50) or depression symptoms (*n* = 40), disruptions to sleep (*n* = 44), increased loneliness (*n* = 38), and increased trauma symptoms (*n* = 15). Five participants specified “other” difficulties/challenges that included: *couldn’t go to church; homelessness; took visits away while incarcerated and at halfway house, so social support is difficult; mandated to wear masks again; going out to places less; not being able to physically get around yourself.* Some women also reported that COVID-19 impacted their mental health in positive ways, including receiving social support from family, friends, partners, or counselors (*n* = 35) or people in the community or local agencies (*n* = 22). Four participants specified other benefits that included: *spend more time with friends/family; isolation (I don’t like to be around a lot of people); learn my husband a little more; having my own transportation.* There were no explanatory variables in the full model of COVID-impact on mental health that met criteria for inclusion in reduced models.

Appendix Table 4 depicts findings from multivariate models of COVID-19-related conflict with partners. Employment status, probable diagnosis of PTSD, cocaine use, and street opioid use were included in reduced models of COVID-19-related conflict with partners. WLWH who were employed experienced less impact of COVID-19 on conflict with their partners than unemployed women (*p* = 0.061). Compared to women who did not meet PTSD criteria, women who met the threshold for PTSD experienced higher impact of COVID-19 on having conflict with their partners (*p* = 0.003). Among women who were using cocaine, compared to those with lower risk use, those who used at moderately/high risk levels experienced greater COVID-19 impact on having conflict with partners (*p* = 0.231). In contrast, compared to those with lower risk use of street opioids, women with moderately/high risk street opioid use experienced lower overall impact of COVID-19 on having conflict with their partners (*p* = 0.153). In the reduced model, only PTSD remained a statistically significant correlate of COVID-19 related conflict with partners.

Some women qualitatively identified positive impacts of the COVID-19 pandemic period on their lives, such as: *stimulus checks, started going back to the gym; had less access to drugs; able to spend more time with family; we look at each other differently now because of it; we talk more than we’ve ever talked; it’s not a good thing to talk about, but it changed our relationship to draw us closer.*

## Discussion

To our knowledge, this study is the first to systematically assess the broad impact of COVID-19 and the experiences of IPV during COVID-19 among WLWH. We did so among a sample of WLWH (mean 53.6 years of age) who had been living with diagnosed HIV for many years (mean 23.3 years), and all of whom had experienced IPV in their lifetimes.

In national surveys, lifetime experience of IPV is relatively common among all U.S. women [1, 40], and even more frequent among WLWH, 55% of whom report IPV exposure [12]. In our cohort of 84 WLWH, all of whom had lifetime IPV exposure, we found higher than expected rates of ongoing IPV. Among 49 women who were currently partnered, 79.6% (*n* = 39) were experiencing ongoing IPV, including physical assault and sexual violence. Findings have important implications for engagement in care, as experiences of IPV among WLWH have been associated with lower levels of treatment adherence and a reduced likelihood of achieving viral suppression [41]. We did not report on HIV treatment outcomes here, which is a primary focus of the ongoing parent study, and these findings will be reported once data collection is complete. Focused research is needed to disentangle the ways in which IPV affects women’s day-to-day experiences of living with HIV, considering potential targets for interventions that support both healthy relationships and engagement in care.

Our findings highlight the many ways in which the COVID-19 pandemic emergency period impacted WLWH who are IPV survivors. Although we do not have a pre-pandemic sample for comparison, the observed high rates of ongoing IPV may reflect increased IPV exposure to WLWH related to “stay at home” regulations [18]. At the height of pandemic-related restrictions in many U.S. settings, HIV care, research participation, and workplace settings transitioned to virtual or telephone-based methods [17]. Participants in this study frequently reported that COVID-19 affected how they received HIV care. Telehealth was critical for the continuous delivery of HIV care during pandemic restrictions, allowing clinics to provide care to highly vulnerable members of the community without compromising health or safety of patients or staff. Yet one inadvertent downside to telehealth was that it may have fostered social isolation, wherein WLWH may have been less able to connect to the community’s critical social and protective networks during the pandemic [17]. Telehealth was also not accessible for many WLWH because of limited health or digital literacy, or limited access to needed technology. Some news reports suggested that telehealth may reduce women’s ability to openly discuss their experiences of IPV (if they choose to do so with their healthcare provider) because of concerns about privacy and risk of their abuser overhearing [5]. We did not address IPV disclosure to healthcare providers via telehealth in this study, though this may be an interesting area for future study.

Though we expected to find that current IPV exposure was associated with more significant COVID-19 impact on various aspects of women’s daily lives, we found no statistically significant differences between those experiencing ongoing IPV and those not experiencing ongoing IPV in terms of demographic characteristics, substance use, mental health, or COVID-19 impact in any domain. We did not find any association between ongoing IPV exposure and COVID-19-related conflict with partners, perhaps because the latter construct was relatively broad and may include relationship stressors that do not meet behavioral threshold of IPV. Alternatively, the impacts of IPV may endure, so that even if these women hadn’t experienced IPV recently, they are still experiencing the impacts of prior experiences, which would make it difficult to see differences between recent and past IPV. Women who had PTSD, however, did experience a greater impact of COVID-19 on having conflict with a partner as compared to women without PTSD.

When we disarticulated the different types of COVID-19 impact domains, we did find additional important correlates of COVID-19 impact. In multivariate linear regression models, we found that COVID-19 impact on physical health was significantly associated with Hispanic ethnicity. Compared to women who were not Hispanic, WLWH who identified as Hispanic reported that COVID-19 had a greater direct effect on their physical health. This finding is consistent with CDC data showing that people who are Hispanic or Latino are 1.5 times more likely to acquire COVID-19, 1.9 times more likely to be hospitalized from COVID-19, and 1.7 times more likely to die from COVID-19 than their non-Hispanic white counterparts [42]. These disparate health outcomes are not because of any biological factor, but rather because of socioeconomic disparities experienced by minoritized communities.

Employment status also was associated with COVID-19 impact on partner conflict, though this was not statistically significant in reduced models. Women who were employed may have experienced changes in their mental health due to shifts to working from home, for those whose jobs allowed them to do so. A previous study showed that working from home was associated with decreased overall mental health due to fewer face-to-face interactions with coworkers, distraction while working, adjusted work hours, and less social support [43]. Working from home may have been particularly stressful for women responsible for parenting with remote schooling, though most of our participants do not have any children under 18 years old living with them. WLWH who were employed were less affected by COVID-19 in terms of conflicts with partners, as compared to unemployed WLWH. This is also consistent with a previous study, in which participants who reported increased conflicts with partners were more likely to be unemployed and less conflict was associated with working part-time [44]. It is unclear whether these findings are related to women’s financial dependency on partners or other factors that may generate stress and conflict within a relationship.

The COVID-19 pandemic brought unique challenges for people with substance use [45]. We found that more severe use of opioids was significantly associated with higher COVID-19 impact on day-to-day life, HIV care, and lower impact on having conflict with partners, whereas more severe use of cocaine had the opposite effects on each domain. These findings are consistent with previous studies showing a rise in substance use and fatal overdoses in the U.S. during the COVID-19 pandemic [46]. People with substance disorders also had increased risk for poor COVID-19 outcomes if they acquired COVID-19 [46]. From an HIV care perspective, untreated substance use disorders are associated with more rapid HIV disease progression, impaired adherence to antiretroviral therapy, and worse overall HIV treatment outcomes [47]. Findings illustrate the importance of addressing and treating substance use disorders to improve substance use outcomes and, secondarily, HIV outcomes. Intervention was particularly important during the pandemic period, when substance use in isolation was associated with high rates of fatal and non-fatal overdose [48]. 

In our sample, mental health problems were associated with a range of COVID-19 impacts. In a recent study of the general U.S. population, nearly half of those surveyed reported recent symptoms of an anxiety or depressive disorder [49]. According to the World Health Organization (WHO), the global prevalence of anxiety and depression has increased 25% since the beginning of the COVID-19 pandemic [50]. Anxiety and depression may be particularly high among WLWH who are IPV survivors. In our analysis, mental health problems, including generalized anxiety, PTSD, and clinically significant depression symptoms were associated with COVID-19 impacts on physical health, mental health, HIV care, and having conflicts with partners, though directionality of this association is not clear. According to WHO, people with pre-existing psychiatric disorders are more likely to experience hospitalization, severe illness and death when they contract COVID-19 than people who do not have psychiatric disorders [50]. People living with HIV (PLWH) report a higher baseline prevalence of psychiatric disorders compared with general population [51]. Especially for WLWH who are IPV survivors, COVID-19 could exacerbate underlying depression, anxiety, and PTSD symptoms, leading to worse mental health outcomes. From an HIV care perspective, we found that WLWH with greater anxiety symptoms experienced a higher impact of COVID-19 on HIV care compared with those who did not have anxiety. This finding is consistent with that from a study of people living with HIV (PLWH) in China, who experienced prolonged lockdowns and isolation [52]. Mental health problems impact HIV care and can worsen health outcomes among PLWH, including decreased medication adherence and viral suppression, and increasing onward HIV transmission risk. Recognition of depression, anxiety, and PTSD symptoms is thus an important priority for WLWH, particularly women who have experienced or are experiencing IPV.

Though novel in its scope and approach, this study is limited by several important factors. First, the results are based on a secondary data analysis from a single point in time. As such, causation cannot be inferred, and any delayed impact of the COVID-19 pandemic and IPV on WLWH was not captured. Second, all measures were self-reported, which can be subject to retrospective and social desirability biases. Third, the sample size was relatively small and geographically confined to a highly resourced setting in New England, and may not reflect the experiences of other WLWH in the U.S. Finally, the sample of WLWH here had all experienced lifetime IPV, so it may have been difficult to tease out associations between types or timing of IPV exposure and COVID-19 impact in this otherwise somewhat homogenous sample.

This study is the first assessment of IPV exposure and COVID-19 impact among WLWH who are IPV-survivors. In this sample, we found high rates of ongoing IPV, and the COVID-19 pandemic affected WLWH in broad and varied ways. This study can inform future strategies to support WLWH who are IPV survivors, which is particularly crucial during emergencies and public health crises.

### Electronic supplementary material

Below is the link to the electronic supplementary material.

Supplementary Material 1

Supplementary Material 2

## Data Availability

The datasets used and/or analyzed during the current study are available from the corresponding author on reasonable request.
