# Supplementary material for: Impact of COVID-19 on women living with HIV who are survivors of intimate partner violence
Source: BMC Public Health. 2024 May 20;24:1352. doi: 10.1186/s12889-024-18862-7 (PMC11103830; doi:10.1186/s12889-024-18862-7)
Supplement: Supplementary file 1 — Supplementary Material 1 [file 12889_2024_18862_MOESM1_ESM.pdf]

COVID-19 Impact

Coronavirus disease 2019 (COVID-19) is a respiratory illness that can spread from person to person. In this section, I will ask you about your experiences with COVID-19. Read each question and select an answer choice below.

How much has COVID-19 directly affected your health, where 1 is not at all up to 5 which is extremely?

- ○ 1 - Not at all
- ○ 2
- ○ 3 - Somewhat
- ○ 4
- ○ 5 - Extremely
- ○ DK
- ○ PS
- ○ DNA

Please tell us which of the following is true about COVID-19 and your health? (Check all that apply)

- ☐ I had symptoms of COVID-19 but did not get tested
- ☐ I was tested for COVID-19 and tested negative
- ☐ I was tested for COVID-19 and tested positive
- ☐ I was exposed to COVID-19
- ☐ I was hospitalized for COVID-19
- ☐ None of the above
- ☐ DK
- ☐ PS
- ☐ DNA

Did you experience discrimination (e.g. people treating you differently) because you were infected with COVID-19? (Check all that apply)

- ☐ No
- ☐ Yes, by people in healthcare settings
- ☐ Yes, by people in the community
- ☐ Yes, by family or close friends
- ☐ Yes, by my partner
- ☐ DK
- ☐ PS
- ☐ DNA

How concerned are you about catching COVID-19?

- ○ Not at all concerned
- ○ A little bit concerned
- ○ Neutral
- ○ Very concerned
- ○ Extremely concerned
- ○ DK
- ○ PS
- ○ DNA

How much has COVID-19 impacted your day-to-day life in general, where 1 is not at all up to 5 which is extremely?

- ○ 1 - Not at all
- ○ 2
- ○ 3 - Somewhat
- ○ 4
- ○ 5 - Extremely
- ○ DK
- ○ PS
- ○ DNA

How much has COVID-19 affected your sexual behaviors, where 1 is not at all up to 5 which is extremely?

- ○ 1 - Not at all
- ○ 2
- ○ 3 - Somewhat
- ○ 4
- ○ 5 - Extremely
- ○ DK
- ○ PS
- ○ DNA

How has COVID-19 affected your sexual behaviors? (Check all that apply)	☐ Have avoided close contact, including kissing and sex, with anyone<br>☐ Only have sex with people I live with<br>☐ Have taken a break from in-person dates<br>☐ Engage in video dates, sexting, or chat rooms<br>☐ Have anal sex more often<br>☐ Use condoms and dental dams more often<br>☐ Wash up before and after sex more often<br>☐ Other (please specify)<br>☐ DK<br>☐ PS<br>☐ DNA
Other (please specify):	__________________________________
How much has COVID-19 affected your substance use, where 1 is not at all up to 5 which is extremely?	◯ 1 - Not at all<br>◯ 2<br>◯ 3 - Somewhat<br>◯ 4<br>◯ 5 - Extremely<br>◯ DK<br>◯ PS<br>◯ DNA
How has COVID-19 affected your substance use? (Check all that apply)	☐ Use more/want to use more<br>☐ Use less/want to use less<br>☐ Stock up on drugs and supplies<br>☐ Prepare my drugs myself<br>☐ Stopped sharing drugs and supplies<br>☐ Plan and prepare for possible overdose (e.g. obtain Narcan for yourself or someone else)<br>☐ Clean surfaces where I prepare drugs, before and after use<br>☐ Wash up before and after drug use more often<br>☐ Started treatment for my drug use or alcohol use<br>☐ Continued treatment for my drug or alcohol use<br>☐ Stopped treatment for my drug or alcohol use<br>☐ Other (please specify)<br>☐ DK<br>☐ PS<br>☐ DNA
Other (please specify):	__________________________________
How much has COVID-19 affected how you use HIV care, where 1 is not at all up to 5 which is extremely?	◯ 1 - Not at all<br>◯ 2<br>◯ 3 - Somewhat<br>◯ 4<br>◯ 5 - Extremely<br>◯ DK<br>◯ PS<br>◯ DNA

How has COVID-19 affected how you use HIV care? (Check all that apply)	☐ Contacted my healthcare provider for guidance about COVID-19<br>☐ Requested a 90-day supply of my HIV medication<br>☐ Started home delivery of my HIV medication<br>☐ Stopped taking my HIV medication<br>☐ Started (or restarted) taking my HIV medication<br>☐ Got my HIV lab/blood testing services done early<br>☐ Cancelled in-person appointment with my doctor due to fear of COVID-19<br>☐ Requested a telehealth visit with my doctor due to fear of COVID-19<br>☐ Other (please specify)<br>☐ DK<br>☐ PS<br>☐ DNA
Other (please specify):	______________________________

Have you experienced any of the following issues with healthcare because of COVID-19? (Select yes or no for each)

	Yes	No	DK	PS	DNA
Clinic closed or doctor cancelled your appointment	○	○	○	○	○

Have you experienced any of the following issues with healthcare because of COVID-19? (Select yes or no for each)

Appointment rescheduled or postponed	○	○	○	○	○

Have you experienced any of the following issues with healthcare because of COVID-19? (Select yes or no for each)

In-person appointment changed to telehealth (phone call or video visit)	○	○	○	○	○

Have you experienced any of the following issues with healthcare because of COVID-19? (Select yes or no for each)

Unable to schedule an appointment	○	○	○	○	○

Have you experienced any of the following issues with healthcare because of COVID-19? (Select yes or no for each)					
Unable to access testing services (i.e. HIV, STD, other)	○	○	○	○	○

Have you experienced any of the following issues with healthcare because of COVID-19? (Select yes or no for each)

Unable to access routine lab/blood testing services	○	○	○	○	○

Have you experienced any of the following issues with healthcare because of COVID-19? (Select yes or no for each)

Unable to get to a pharmacy	○	○	○	○	○

Have you experienced any of the following issues with healthcare because of COVID-19? (Select yes or no for each)

Unable to get prescription(s) filled/refilled	○	○	○	○	○

Have you experienced any of the following issues with healthcare because of COVID-19? (Select yes or no for each)

Loss of health insurance	○	○	○	○	○

Have you experienced any of the following issues with healthcare because of COVID-19? (Select yes or no for each)

Other (please specify)	◯	◯	◯	◯	◯

Other healthcare issues (please specify) __________________________________

How much has COVID-19 affected your mental or emotional health (in either positive or negative ways), where 1 is not at all up to 5 which is extremely?

- ◯ 1 - Not at all
- ◯ 2
- ◯ 3 - Somewhat
- ◯ 4
- ◯ 5 - Extremely
- ◯ DK
- ◯ PS
- ◯ DNA

Have you experienced any of the following issues related to your mental or emotional health due to COVID-19? (check all that apply)

- ☐ Frustration or boredom
- ☐ More anxiety than usual
- ☐ Less anxiety than usual
- ☐ More depression than usual
- ☐ Less depression than usual
- ☐ More sleep, less sleep, or other changes to your normal sleep pattern
- ☐ Loneliness
- ☐ Trauma symptoms (flashbacks, nightmares)
- ☐ Received social support from family, friends, partners, a counselor, or someone else
- ☐ Received social support from people in your community or local agencies
- ☐ Other difficulties or challenges (specify)
- ☐ Other benefits (specify)
- ☐ DK
- ☐ PS
- ☐ DNA

Other difficulties and challenges (please specify) __________________________________

Other benefits (please specify) __________________________________

Challenges and Hardships

How much has COVID-19 affected your financial or economic status, where 1 is not at all up to 5 which is extremely?

- ◯ 1 - Not at all
- ◯ 2
- ◯ 3 - Somewhat
- ◯ 4
- ◯ 5 - Extremely
- ◯ DK
- ◯ PS
- ◯ DNA

Have you experienced any of the following issues related to your financial or economic situation due to COVID-19? (check all that apply)

- ☐ Loss of employment
- ☐ Loss of income
- ☐ Had to ask for financial support from family, friends, partners, an organization, or someone else
- ☐ Not having enough basic supplies (e.g., food, water, clothing, shelter)
- ☐ Difficulty getting my medications
- ☐ Difficulty getting financial support from family, friends, partners, an organization, or someone else
- ☐ Forced to change housing situation
- ☐ Loss of transportation (reductions in public transit services, loss of ride-sharing due to fear of Coronavirus exposure)
- ☐ Loss of health insurance coverage
- ☐ Had to draw on savings
- ☐ Other difficulties or challenges (specify):
- ☐ DK
- ☐ PS
- ☐ DNA

Other difficulties or challenges (please specify)

____

How much has COVID-19 affected your experience with conflicts with [bdem17b], where 1 is not at all up to 5 which is extremely?

- ◯ 1 - Not at all
- ◯ 2
- ◯ 3 - Somewhat
- ◯ 4
- ◯ 5 - Extremely
- ◯ DK
- ◯ PS
- ◯ DNA

How have conflicts with [bdem17b] changed during COVID-19?

- ◯ I've experienced more conflict/fights than usual
- ◯ I've experienced less conflict/fights than usual
- ◯ Other (please specify)
- ◯ DK
- ◯ PS
- ◯ DNA

Other conflict related changes with [bdem17b] (please specify)

____

Positive Impact

Have there been any positive changes in your life that were a result of COVID-19?

◯ Yes
◯ No
◯ DK
◯ PS
◯ DNA

What is the positive thing in your life that has changed as a result of COVID-19?

____

Have you received the COVID-19 vaccine?

◯ Yes, I have received the first of 2 doses
◯ Yes, I have received the second of 2 doses, or received dose 1 of 1
◯ No but I have a scheduled appointment to get the vaccine
◯ Not yet but I plan to get the vaccine
◯ Not yet and I don't plan to get the vaccine
◯ I have not made a decision on whether or not I will get the vaccine
