# Supplementary material for: Impact of COVID-19 on women living with HIV who are survivors of intimate partner violence
Source: BMC Public Health. 2024 May 20;24:1352. doi: 10.1186/s12889-024-18862-7 (PMC11103830; doi:10.1186/s12889-024-18862-7)
Supplement: Supplementary file 2 — Supplementary Material 2 [file 12889_2024_18862_MOESM2_ESM.docx]

**Appendix Tables**

For all Tables below, full models of COVID-19 impact included current IPV exposure, age, race, ethnicity, years of education, employment status, PTSD, anxiety, depression, and substance use (alcohol, cannabis, cocaine, opioids). Only variables with p-value <0.2 in the full model are included in the reduced model and shown here; all other covariates had no statistically significant correlations with the outcome of interest.

**Appendix Table 1. Factors associated with COVID-impact on physical health**

| Characteristic | Full Model (N=84) | | Reduced Model (N=84) | |
| --- | --- | --- | --- | --- |
|  | Beta (SE) | p-value | Beta (SE) | p-value |
| Ethnicity |  |  |  |  |
| Non-Hispanic | Reference |  | Reference |  |
| Hispanic | 0.930 (0.500) | 0.067 | 0.927 (0.334) | 0.007 |
| Anxiety  No generalized anxiety | Reference |  | Reference |  |
| Generalized anxiety | -0.675 (0.451) | 0.139 | -0.537 (0.364) | 0.144 |
| Depression |  |  |  |  |
| No depression | Reference |  | Reference |  |
| Clinically significant depression | 1.102 (0.397) | 0.007 | 1.14 (0.320) | 0.0006 |

**Appendix Table 2. Factors associated with COVID-impact on day-to-day life**

| Characteristic | Full Model (N=84) | | Reduced Model (N=84) | |
| --- | --- | --- | --- | --- |
|  | Beta (SE) | p-value | Beta (SE) | p-value |
| Race  Black/African American  White | Reference  -1.202 (0.559) | 0.035 | Reference  -0.922 (0.454) | 0.046 |
| Cocaine  Lower risk  Moderate/high risk | Reference  -0.876 (0.452) | 0.057 | Reference  -0.731 (0.407) | 0.076 |
| Street opioids  Lower risk  Moderate/high risk | Reference  1.072 (0.579) | 0.068 | Reference  1.124 (0.489) | 0.024 |
| Prescription opioids  Lower risk  Moderate/high risk | Reference  1.425 (0.651) | 0.032 | Reference  1.291 (0.566) | 0.025 |

**Appendix Table 3. Factors associated with COVID-impact on HIV care**

| Characteristic | Full Model (N=84) | | Reduced Model (N=84) | |
| --- | --- | --- | --- | --- |
|  | Beta (SE) | p-value | Beta (SE) | p-value |
| Race  Black/other  White | Reference  -0.885 (0.497) | 0.079 | Reference  -0.733 (0.396) | 0.068 |
| Years of Education | 0.119 (0.089) | 0.182 | 0.126 (0.079) | 0.116 |
| Anxiety  No generalized anxiety | Reference |  | Reference |  |
| Generalized anxiety | 0.900 (0.472) | 0.061 | 0.714 (0.325) | 0.030 |
| Cocaine  Lower risk  Moderate/high risk | Reference  -0.613 (0.402) | 0.132 | Reference  -0.574 (0.354) | 0.109 |
| Street opioids  Lower risk  Moderate/high risk | Reference  0.694 (0.515) | 0.182 | Reference  0.904 (0.441) | 0.044 |

**Appendix Table 4. Factors associated with COVID-impact on having conflict with partner**

| Characteristic | Full Model (N=48) | | Reduced Model (N=48) | |
| --- | --- | --- | --- | --- |
|  | Beta (SE) | p-value | Beta (SE) | p-value |
| Employment |  |  |  |  |
| Not employed | Reference |  | Reference |  |
| Employed | -1.505 (0.805) | 0.071 | -1.169 (0.606) | 0.061 |
| PTSD  No PTSD | Reference |  | Reference |  |
| PTSD | 0.849 (0.570) | 0.146 | 1.318 (0.420) | 0.003 |
| Cocaine  Lower risk  Moderate/high risk | Reference  0.934 (0.651) | 0.161 | Reference  0.589 (0.484) | 0.231 |
| Street opioids  Lower risk  Moderate/high risk | Reference  -1.111 (0.675) | 0.110 | Reference  -.776 (0.533) | 0.153 |
